# Supplementary material for: Lamin Mutations Cause Increased YAP Nuclear Entry in Muscle Stem Cells
Source: Cells. 2020 Mar 27;9(4):816. doi: 10.3390/cells9040816 (PMC7226749; doi:10.3390/cells9040816)
Supplement: Supplementary file 1 [file cells-09-00816-s001.pdf]

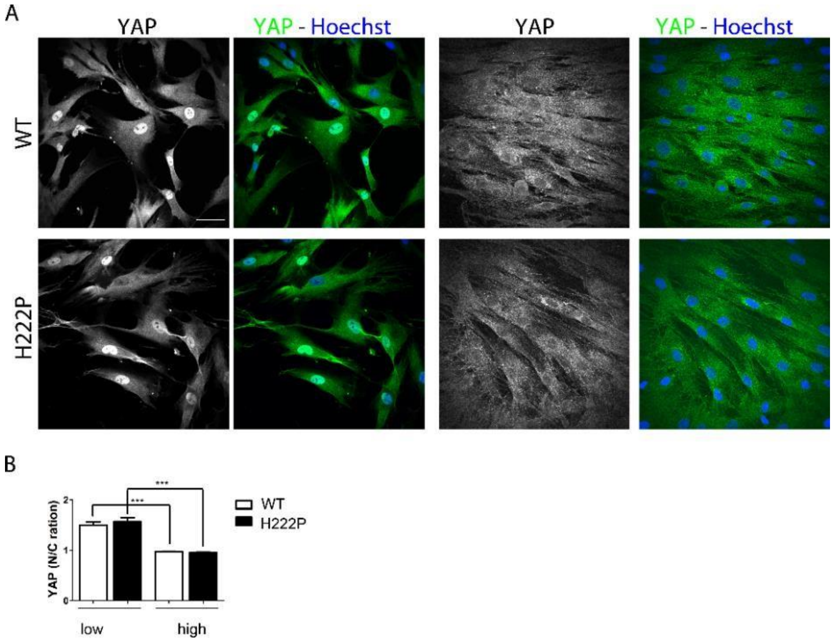

Figure S1. A) Confocal images of YAP (green) in confluent WT and *LMNA*$^{\mathrm{H222P}}$ myogenic cells cultured in low and high density conditions. Nuclei are stained with Hoechst (blue). Scale bar: 50 µm. C) Quantification of YAP nucleo-cytoplasmic (N/C) ratio. Values are expressed as mean ± SEM, n=50 in WT and 55 in *LMNA*$^{\mathrm{H222P}}$ cell lines.

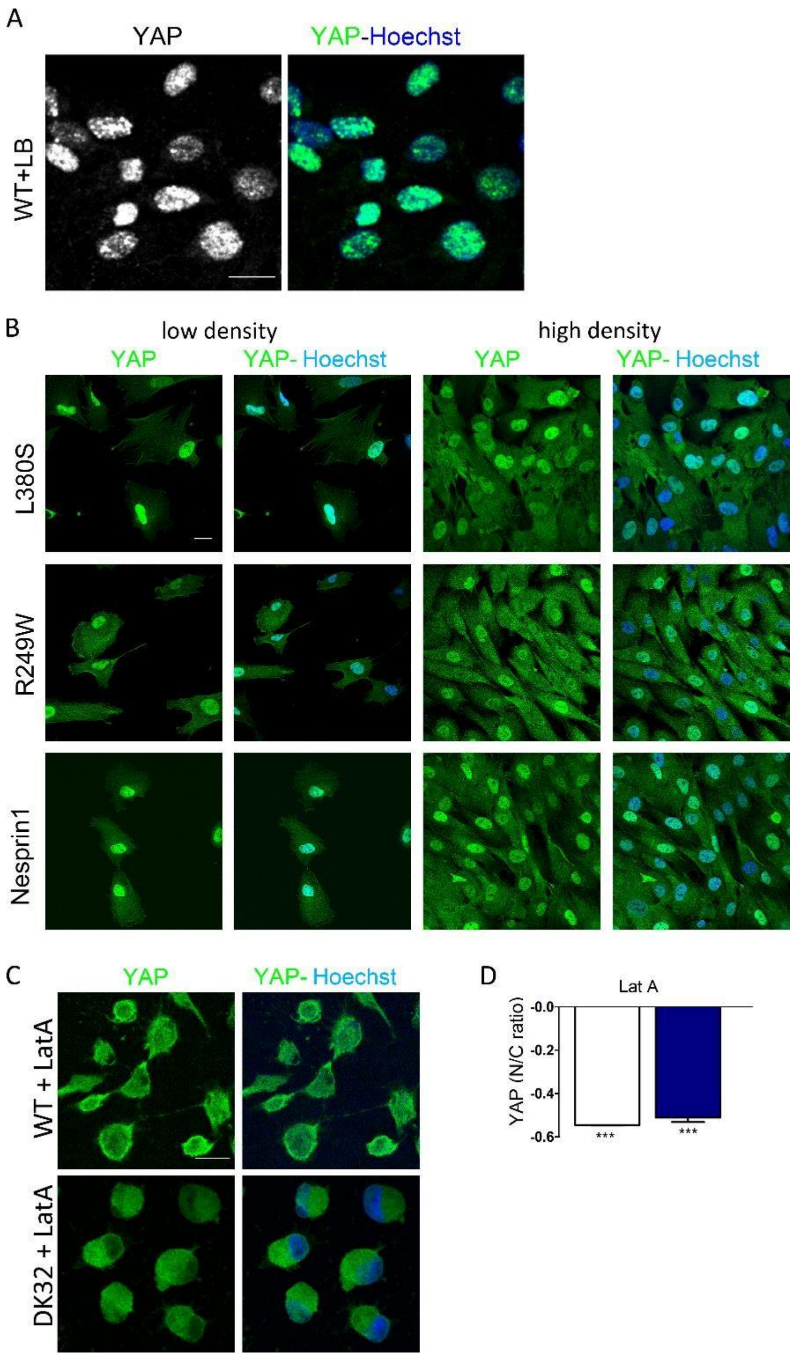

Figure S2. A) Confocal images of YAP (green) and nuclei (blue) in confluent WT MuSCs treated with Leptomycin B (LB). Scale bar=20µm; B) Confocal images of YAP (green) and nuclei (blue) in WT and *LMNA*$^{\Delta K32}$ mutant MuSCs cultured in low and high density conditions. Nuclei are stained with Hoechst (blue). Scale bar: 20 µm. C) Confocal images of YAP (green) and nuclei (blue) in confluent WT MuSCs treated with LatA. Scale bar=20µm; D) Quantification of YAP nucleo-cytoplasmic (N/C) ratio after latrunculin (Lat A) treatment expressed as a fraction of control value obtained in sparse conditions. Values are expressed as mean ± SEM, n=100 cells for each cell line, ***p<0.001 *versus* value obtained before LatA treatment.

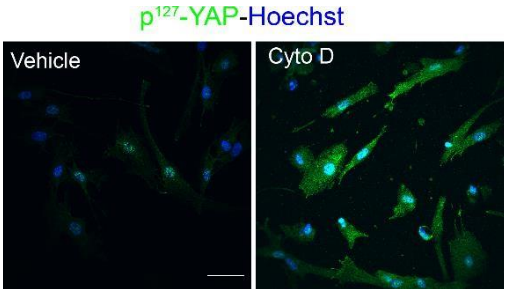

Figure S3. Confocal images of p^{127}-YAP (green) and nuclei (blue) in WT MuSCs treated with vehicle (DMSO) or Cytochalasin D (cyto D). Scale bar = 50 µm.

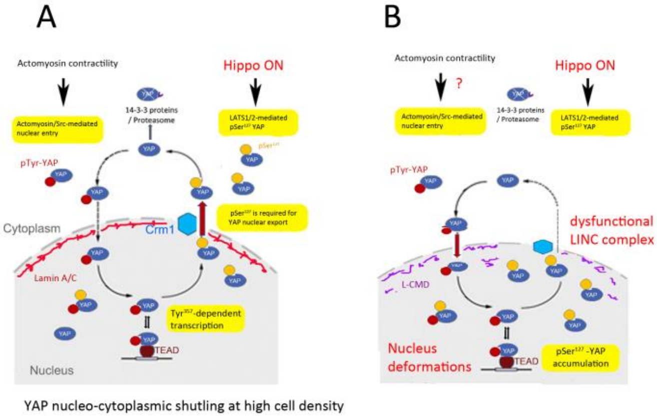

Figure S4. Proposed model of the mechanisms by which lamins influence YAP subcellular distribution at high cell density. A) In WT MuSCs plated at high cell density, the Hippo pathway is activated and p^{127}-YAP was excluded from the nucleus. YAP nuclear entry was lower than YAP nuclear export. B) In L-CMD and nesprin-1$^{\Delta KASH}$ mutations, YAP nuclear export is insufficient to counterbalance YAP nuclear entry despite activation of the Hippo pathway. This results in persistent nuclear localization of p^{S127}-YAP nuclear import. Potential mechanisms imply increased nuclear deformability and/or dysfunctional LINCcomplex.
